# Predictors for influenza vaccination among Thai pregnant woman: The role of physicians in increasing vaccine uptake

**DOI:** 10.1111/irv.12674

**Published:** 2019-08-16

**Authors:** Surasak Kaoiean, Wanitchaya Kittikraisak, Piyarat Suntarattiwong, Darunee Ditsungnoen, Podjanee Phadungkiatwatana, Nattinee Srisantiroj, Suvanna Asavapiriyanont, Tawee Chotpitayasunondh, Fatimah S. Dawood, Kim A. Lindblade

**Affiliations:** ^1^ Rajavithi Hospital Ministry of Public Health Bangkok Thailand; ^2^ Influenza Program Thailand Ministry of Public Health – U.S. Centers for Disease Control and Prevention Collaboration Nonthaburi Thailand; ^3^ Queen Sirikit National Institute of Child Health Ministry of Public Health Bangkok Thailand; ^4^ Influenza Division U.S. Centers for Disease Control and Prevention Atlanta GA USA

**Keywords:** influenza, physician, predictor, pregnant woman, Thailand, vaccination

## Abstract

**Background:**

Physician recommendation and attitudes and beliefs of pregnant women toward influenza and vaccination may influence vaccine uptake during pregnancy. We examined how physician recommendation and health beliefs of pregnant women may jointly affect influenza vaccination during pregnancy.

**Methods:**

Thai pregnant women aged ≥18 years and >13 gestational weeks attending antenatal care (ANC) clinics, and ANC physicians were recruited during May‐August 2015. Women and physicians, linked using unique identifiers, provided data on demographic, health and work history, knowledge, attitudes, and beliefs toward influenza and vaccination, based on Health Belief Model constructs. Physicians also provided data on their practices in recommending influenza vaccination during pregnancy. Prevalence ratios for the association between knowledge, attitudes and beliefs of pregnant women, physician recommendation and documented receipt of vaccination within 30 days of the visit were calculated.

**Results:**

Among 610 women, the median age was 27 years; 266 (44%) and 344 (56%) were in the second and third trimesters, respectively. Twenty‐one (3%) had pre‐existing conditions. Of 60 physicians with the median years of practice of 5; 17 (28%) reported frequently/usually/always recommending influenza vaccine to their pregnant patients, while 43 (72%) reported never/rarely/sometimes recommending the vaccine. Controlling for the pregnant women's knowledge and beliefs, pregnant women whose physician recommended influenza vaccination were 2.3 times (95% confidence interval 1.4‐3.8) more likely to get vaccinated.

**Conclusions:**

In this study, physician recommendation was the only significant factor associated with influenza vaccine uptake among Thai pregnant women. Understanding physicians’ motivation/barrier to recommending influenza vaccination to pregnant women may increase coverage.

## 1 BACKGROUND

Pregnant women are at increased risk of hospitalization due to influenza compared with both the general population and women of childbearing age,^1^, ^2^ although there is inconsistent and limited evidence that influenza virus infection causes adverse pregnancy outcomes.^3^ While some studies have reported little or no increased risk of preterm birth among women who were hospitalized with influenza during pregnancy,^4^, ^5^ others found an approximately four‐fold increased risk of preterm birth.^6^, ^7^ Influenza vaccination during pregnancy effectively prevents infection among pregnant women and confers protection to their infants during the first few months of life.^8^, ^9^, ^10^, ^11^, ^12^, ^13^, ^14^ A study has shown the influenza vaccine also provided low‐to‐moderate protection against hospitalization with influenza virus infection.^2^ Reductions of 13% of preterm births and 26% of low birth weight were reported,^8^, ^9^, ^15^ but no effect on small for gestational age was reported.^16^ The safety of influenza vaccination during pregnancy was determined through post‐licensure vaccine safety monitoring platforms: pregnancy exposure registries; active and passive surveillance systems; and observational studies.^17^ These platforms have demonstrated the safety of influenza vaccine administration during pregnancy to the mother, fetus, and newborn^18^, ^19^ and few non‐medically and medically attended adverse events.^20^, ^21^, ^22^ They also demonstrated no increase in pregnancy complications^23^, ^24^, ^25^, ^26^, ^27^, ^28^, ^29^, ^30^, ^31^, ^32^ and no adverse effects following first trimester administration of vaccine.^28^, ^30^, ^33^

Pregnant women are considered a priority group for influenza vaccination by the World Health Organization, but hesitancy of both physicians and pregnant women about the safety of medical interventions during pregnancy may be a barrier to increasing vaccination coverage rates. A recent population‐based survey in China has reported only 11% of pregnant women reported being willing to receive an influenza while pregnant.^34^ The same survey also indicated that only 20% of the obstetricians being willing to recommend influenza vaccination to their pregnant women. Other study has shown a systematic different in socio‐demographic and health characteristics of those who decided to get vaccinated with an influenza vaccine.^35^ In a recent cluster randomized trial, a multimodal intervention has been shown to effectively increase influenza vaccination in the USA’s obstetrics/gynecology settings,^36^ although a review of published studies through mid‐2015 of factors influencing vaccination during pregnancy in which 73% of studies addressed influenza vaccination found that concerns about vaccine safety, beliefs that vaccination was unnecessary, poor knowledge about vaccines or disease, absence of physician recommendation, and limited vaccine access were barriers to vaccination.^37^ Inadequate reimbursement, lack of training, and increased workload were the main barriers to recommending influenza vaccination by physicians. Sixty‐five percent of studies related to influenza vaccination were from North America, and 81% of all studies focused on pregnant women's perceptions of vaccination with relatively few studies focused on provider's attitudes, beliefs, and practices. Few studies have simultaneously evaluated physicians’ and pregnant woman's attitudes and beliefs about influenza vaccination during pregnancy to determine the impact of the interaction between physicians and vaccine recipients on vaccination uptake.^37^, ^38^ We examined how these two perspectives may affect influenza vaccination during pregnancy.

## 2 METHODS

### 2.1 Study design and setting

During May‐August 2015, we conducted a cohort study of pregnant women attending the antenatal care (ANC) clinic and the physicians at the ANC of Rajavithi Hospital (Bangkok, Thailand), a 1200‐bed facility that provides ANC services to an average of 137 Thai pregnant women daily. The Thai Ministry of Public Health (MOPH) provides limited doses of influenza vaccine free of charge nationwide, from May through August, to all risk groups including pregnant women in the second or third trimester of pregnancy through the annual campaign on the first come first served basis.

During May‐July 2015, the hospital strengthened its influenza vaccination program by relocating the influenza vaccination services to the ANC area, providing educational seminars to ANC staff, including physicians, about the benefits and safety of influenza vaccination, and increasing the communication materials posted around the ANC clinic. These efforts were undertaken because the rate of influenza vaccination among pregnant women at Rajavithi Hospital was low (~0.1%) before study initiation, and to assist with preparations for a cohort study of the impact of influenza vaccination on birth outcomes. At the time this study began, influenza vaccination coverage had increased to approximately 13%.

### 2.2 Enrollment of pregnant women and data collection

At the reception desk of the ANC clinic, study nurses screened pregnant women for eligibility according to information on appointment slips. Study nurses then systematically approached presumed eligible women while they waited for consultations to confirm eligibility. Eligible participants were ≥18 years old, Thai citizens, and in the second or third trimester of pregnancy.

Following written informed consent, study nurses administered a structured questionnaire to collect demographic and health history data from the participants before the physician consultation. Study nurses did not participate in the consultations; decision to discuss or recommend any vaccination was left to the physicians. After each consultation, study nurses added unique codes to the women's records to identify the attending physicians. Participants completed the second part of the questionnaire requesting information on the knowledge (Table S1), attitudes and beliefs toward influenza illness and vaccination. To ensure correct understanding of the questions while minimizing socially acceptable response bias and distractions from the busy ANC clinic, participants used an audio, computer‐assisted self‐interview (ACASI) data collection method with headphones and a computer tablet.^39^ Study nurses were available to answer any questions the participants may have had during the self‐interview.

### 2.3 Enrollment of physicians and data collection

A list of physicians who regularly provided care at ANC clinic was obtained. Physicians, excluding study investigators (n = 4), who had served ≥6 months in the Obstetrics and Gynecology department of Rajavithi Hospital were eligible for inclusion. Following written informed consent, physicians self‐completed a questionnaire on a computer tablet requesting information on demographics, work history, knowledge (Table S1), attitudes, and beliefs toward influenza vaccination in pregnant women, and their regular practice regarding recommending influenza vaccination to pregnant women.

### 2.4 Data collection instruments

Questions on attitudes, perceptions, and beliefs about influenza and vaccination were based on the Health Belief Model (HBM) with five constructs that influence health behaviors: perceptions of susceptibility to disease; perceptions of severity of disease; barriers to preventive behaviors; perceived benefits of preventive behaviors; and cues to action.^40^ Questions were adapted from published literature for influenza vaccination^41^, ^42^, ^43^, ^44^ and translated into the Thai language (Table S2) and pilot tested among a sample of Thai pregnant women. We used a similar set of questions to examine physician's beliefs about influenza in pregnancy, perceptions of vaccination, and cues to action (Table S3).

Physicians were asked to describe their frequency in recommending the influenza vaccine to pregnant women at Rajavithi Hospital during the previous six months. Responses were grouped by physicians’ reported practice: usually, frequently, or always (≥50% of eligible patients; “frequent recommenders”) or never, rarely, occasionally, or sometimes (<50% of eligible patients; “non‐frequent recommenders”). Physicians were asked whether they knew of and agreed with the MOPH policy to vaccinate pregnant women against influenza after the first trimester.

The study primary outcome, verified from medical records, was documented the receipt of seasonal influenza vaccine within 30 days of the ANC visit among participating women who had not previously been vaccinated during the current pregnancies. Vaccination up to 30 days after the ANC visit was included as an outcome because hospital practice did not allow for tetanus and influenza vaccine to be given at the same visit.

### 2.5 Sample size

We calculated that 620 pregnant women would be needed to detect a prevalence ratio of 2.0 for the association between beliefs about influenza vaccine and receipt of vaccine, assuming a ≥15% vaccination rate, 25% of the women held a certain health belief, power of 80%, and the probability of a Type I error of 5%.^45^ Assuming 10% of pregnant women attending the Rajavithi Hospital's ANC clinic were in the first trimester and 15% refusal rate, 827 women would need to be approached for enrollment.

Because of the limited number of physicians at the ANC clinic, all were screened for eligibility and invited to participate.

### 2.6 Data analysis

Responses to the questions on health beliefs, attitudes, and beliefs were grouped according to the matching HBM construct. For the purpose of analysis, responses were coded such that higher values represented higher levels of the HBM construct (eg, a high score on susceptibility to influenza indicated the respondent felt highly susceptible to influenza, whereas a high score on barriers to vaccination indicated that the respondent identified a high level of barriers to vaccination). The scores of respondents’ answers to all questions within each construct were summed and used as their score for that construct. The total score of each construct was divided into two (low and high) or three categories (low, moderate, and high), using SAS Proc Rank (SAS 9.3, SAS Institute) depending on the distribution of responses (Table S4).

For analyzes of the association between pregnant women's receipt of vaccination and physicians’ and women's characteristics and beliefs, we excluded pregnant women whose physicians declined to participate or served as study investigators and pregnant women reporting influenza vaccination before enrollment. Demographic characteristics and knowledge, attitudes, and beliefs were compared between pregnant women who did and did not receive an influenza vaccination using the chi‐square test for categorical variables and the non‐parametric Kruskal‐Wallis test for continuous variables. Similarly, characteristics of frequent and non‐frequent recommenders were compared. *P* values < .05 were considered statistically significant.

To measure the association between physician recommendation and the HBM constructs for pregnant women and vaccination, a generalized estimating equations approach with a binary distribution, log link, and an exchangeable correlation structure to account for the correlation due to multiple patients cared for by the same physician was used to estimate prevalence ratios. All factors found significantly associated with vaccination in the univariate analysis were entered into a multivariable model, and interaction term was evaluated (perceptions of the benefits of influenza vaccination and physician recommendation). Adjusted prevalence ratios and 95% confidence intervals (CI) were calculated. *P* values < .05 were considered statistically significant.

### 2.7 Ethical review

The study was approved by the Ethical Review Committees for Research in Human Subjects of the Thai MOPH (MOPH ERC, Bangkok, Thailand) and Rajavithi Hospital (Bangkok, Thailand). The US Centers for Disease Control and Prevention's Institutional Review Board (Atlanta, USA) relied on the MOPH ERC’s determination.

## 3 RESULTS

### 3.1 Pregnant women

Between May and August 2015, we systematically approached 839 (34%) of 2,458 pregnant women presumed to be eligible. Of these, 835 (99%) were eligible for study participation; 620 (74%) agreed to participate. Among consenting women, 610 (98%) completed the two‐step data collection process. The median age was 27 years (interquartile range [IQR], 23‐33; Table S5). There were 266 (44%) and 344 (56%) women in the second and the third trimester, respectively. Twenty‐one (3%) women had pre‐existing conditions. The most common pre‐existing conditions were liver disease (seven women) and hemoglobinopathy (five women). Four hundred and thirty‐two (71%) were employed outside the home, 208 (34%) had household income <30 000 Baht/month (876 US dollars), and 428 (70%) had completed secondary school education or higher. Only 206 (34%) women correctly answered at least nine out of 11 questions on symptoms, modes of transmission, and treatment of influenza, benefits of vaccination, and high‐risk groups recommended for influenza vaccination. There were 515 (84%) women who knew of the Thai MOPH policy for free influenza vaccination during pregnancy, and 521 (85%) had seen promotional material for the influenza vaccination that was posted at the ANC clinic during the enrollment visit. Eighty‐nine (15%) women stated they had received influenza vaccination before study enrollment; of which 87 were verified through medical records as vaccinated in the concurrent year.

There were 136 (22%) of women who perceived their susceptibility to influenza was high, with the majority (474, 78%) rating their own susceptibility as moderate or low (Table S4). However, most women perceived the benefits of vaccination (343, 56%) and the potential severity of illness (435, 71%) to be high. Most women (484, 79%) perceived the barriers to vaccination to be moderate or low. A majority of pregnant women (329, 54%) reported readiness to get vaccinated if suggested by physician/family member or if workplace provided influenza vaccine (eg, high level of cues to action).

### 3.2 Physicians

**Figure 1:**
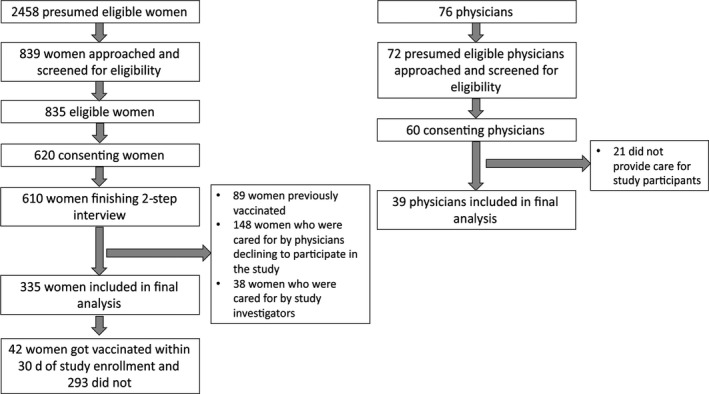
Flow diagram of pregnant women and healthcare providers participating in the study

During the enrollment period, there were 76 physicians at the ANC clinic. We excluded four physicians who were also study investigators, leaving 72 physicians to be approached for enrollment (Figure 1). Among these, 60 (83%) agreed to participate; 39 of whom provided care to study participants while 21 provided care to other pregnant women but study participants. The median age of 60 physicians was 30 years (IQR 28‐33; Table S6). The majority (50, 83%) were female with a median number of five years in practice (IQR 4‐8). Forty‐one (68%) physicians had a high level of knowledge of influenza (ie, scored at least nine correct answers out of 11 questions asked on symptoms, modes of transmission, and treatment of influenza, benefits of vaccination, and high‐risk groups recommended forvinfluenza vaccination). All (100%) physicians were aware of the MOPH policy to vaccinate pregnant women for influenza and 53 (88%) agreed with the MOPH policy to vaccinate pregnant women for influenza. Seventeen (28%) physicians stated that they were frequent recommenders, and 43 (72%) were non‐frequent recommenders. Fifty‐three (88%) physicians reported having ever received influenza vaccination themselves.

The majority of physicians (54, 90%) perceived the susceptibility of pregnant women to influenza illness to be high, and more than three‐quarters (46, 77%) thought the benefits of influenza vaccination to pregnant women and their fetuses to be high. Similarly, 47 (78%) physicians thought pregnant women had an increased risk of getting severely ill from influenza compared with other women. There were 13 (22%) physicians who perceived the barriers to vaccination for pregnant women were low, 24 (40%) who perceived the barriers to be moderate, and 23 (38%) who perceived the barriers to be high.

### 3.3 Predictors of vaccination

For analyzes of the impact of the interaction between physician and patient on vaccination, we excluded 89 women with reported influenza vaccination before enrollment, 148 women who were cared for by physicians declining to participate, and 38 women under the care of study investigators, resulting in an analytical sample of 335 pregnant women (Figure 1). There were no significant differences in characteristics, knowledge, attitudes, or beliefs between the women included in the analysis and those not included (data not shown). The women in the analytical sample were cared for by 39 out of the 60 participating physicians. The median number of pregnant women cared for by these 39 physicians was 5 women (IQR, 3‐9).

We identified 43 (13%) pregnant women who received an influenza vaccination. Of whom, 42 (98%) received the vaccine within 30 days of their ANC visits and 1 (2%) at 35 days following her ANC visit. Of those receiving the vaccine within 30 days, the median time from physician consultation to influenza vaccination was 0 day (IQR, 0‐14). Vaccinated and unvaccinated women were generally comparable in terms of age, trimester, pre‐existing condition, employment status, household income, education, knowledge of influenza, and whether they had seen promotional material at or before enrollment visit (Table 1). However, vaccinated women were significantly more likely than unvaccinated women to know of the MOPH policy on influenza vaccination for pregnant women (95% vs. 82%, *P* value = .03) and to have received a physician recommendation of influenza vaccination at their ANC visit (69% vs. 38%, *P* value < .01). There were no statistically significant differences in HBM constructs between vaccinated and unvaccinated pregnant women.

**Table 1:** Characteristics and Health Belief Model constructs of pregnant women who were vaccinated compared with those who were not

Variables	Vaccinated pregnant women (N = 42) n (%)	Unvaccinated women (N = 293) n (%)	*P* value
Age, median (IQR)	25 (22‐33)	28 (23‐34)	.26
Trimester			
Second	25 (60)	147 (50)	.26
Third	17 (40)	146 (50)	
Had pre‐existing condition	0 (0)	17 (6)	.11
Employed outside home	26 (62)	187 (64)	.81
Household income <30 000 Baht/month (876 US dollars)^a^	29 (69)	192 (66)	.74
Completed secondary school or higher	32 (76)	250 (85)	.13
High level knowledge of influenza^b^	17 (40)	91 (31)	.22
Knew MOPH policy on influenza vaccination of pregnant women	40 (95)	239 (82)	.03
Saw promotional material at or before enrollment visit	36 (86)	245 (84)	.73
Physician recommended influenza vaccine	29 (69)	111 (38)	<.01
Health Belief Model constructs			
Susceptibility to influenza			
High	6 (14)	62 (21)	.58
Moderate	18 (43)	113 (39)	
Low	18 (43)	118 (40)	
Benefits of influenza vaccination			
High	26 (62)	157 (54)	.06
Moderate	11 (26)	52 (18)	
Low	5 (12)	84 (29)	
Severity of influenza illness			
High	32 (76)	199 (68)	.28
Low	10 (24)	94 (32)	
Barriers to influenza vaccination			
High	5 (12)	76 (26)	.13
Moderate	27 (64)	163 (56)	
Low	10 (24)	54 (18)	
Cues to action to influenza vaccination			
High	24 (57)	146 (50)	.38
Low	18 (43)	147 (50)	

Abbreviations: IQR, interquartile range; MOPH, Ministry of Public Health.

a Among those who answered this question.

b Scored at least 9 correct answers out of 11 questions asked on symptoms, modes of transmission, and treatment of influenza, benefits of vaccination, and high‐risk groups recommended for influenza vaccination.

We found no statistically significant differences in demographic characteristics, or knowledge, attitudes, and beliefs toward influenza and vaccination between frequent and non‐frequent recommenders (Table 2). All (13 of 13; 100%) of frequent recommenders and 21 of 26 (81%) non‐frequent recommenders agreed with the MOPH policy on influenza vaccination during pregnancy. From the itemized health belief questions, frequent recommenders tended to be less concerned about vaccine safety in pregnant women than non‐frequent recommenders (77% vs. 100%; *P* value = .08) and the developing fetuses (77% vs. 100%; *P* value = .08; Table S3). Among all cues to action, physicians in both groups reported relying on policies for influenza vaccination in pregnancy from the MOPH (92% vs. 85%; *P* value = .53) and academic society (100% vs. 85; *P* value = .14) than that of the hospital (0% vs. 4%; *P* value = .46). More than half of frequent recommenders (7 of 13; 54%) and non‐frequent recommenders (17 of 26; 65%) concerned about adverse effects from an influenza vaccine in pregnant women. Additionally, about one third of frequent recommenders (4 of 13; 31%) and non‐frequent recommenders (9 of 26; 35%) stated that amount of time needed for consultation with their pregnant patients was an important consideration when deciding whether to recommend influenza vaccine.

**Table 2:** Characteristics and Health Belief Model constructs of physicians by frequency of recommending influenza vaccine to pregnant women

Variables	Frequent recommenders^a^ (N = 13) n (%)	Non‐frequent recommenders^a^ (N = 26) n (%)	*P* value
Age, median (IQR)	29 (28‐32)	31 (29‐38)	.20
Years of practice, median (IQR)	5 (4‐8)	5 (4‐12)	.67
Female	11 (85)	20 (77)	.57
High level of knowledge of influenza^b^	8 (62)	15 (58)	.82
Agreed with MOPH policy on influenza vaccination of pregnant women	13 (100)	21 (81)	.09
Health Belief Model constructs regarding perceptions of pregnant women			
Susceptibility to influenza			
High	11 (85)	23 (88)	.73
Low	2 (15)	3 (12)	
Benefits of influenza vaccination			
High	11 (85)	17 (65)	.21
Low	2 (15)	9 (35)	
Severity of influenza illness			
High	12 (92)	19 (73)	.16
Low	1 (8)	7 (27)	
Barriers to vaccination			
High	4 (31)	12 (46)	.47
Moderate	5 (38)	10 (39)	
Low	4 (31)	4 (15)	
Cues to action to recommend influenza vaccination			
High	12 (92)	20 (77)	.39
Low	1 (8)	6 (23)	

Abbreviations: IQR, interquartile range; MOPH, Ministry of Public Health.

a Frequent recommenders were those reported recommending influenza vaccination to ≥50% of eligible pregnant women; non‐frequent recommenders were those reported recommending influenza vaccination to <50% of eligible pregnant women.

b Scored at least 9 correct answers out of 11 questions asked on symptoms, modes of transmission, and treatment of influenza, benefits of vaccination, and high‐risk groups recommended for influenza vaccination.

We compared physicians’ self‐reported frequency of vaccine recommendation to reports from pregnant women of whether they received a physician recommendation for influenza vaccine. Forty‐nine of 121 (41%) women whose physicians were frequent recommenders received a recommendation for influenza vaccine during their ANC visit, compared with 91 of 214 women (43%) whose physicians were non‐frequent recommenders (*P* value = .70).

**Figure 2:**
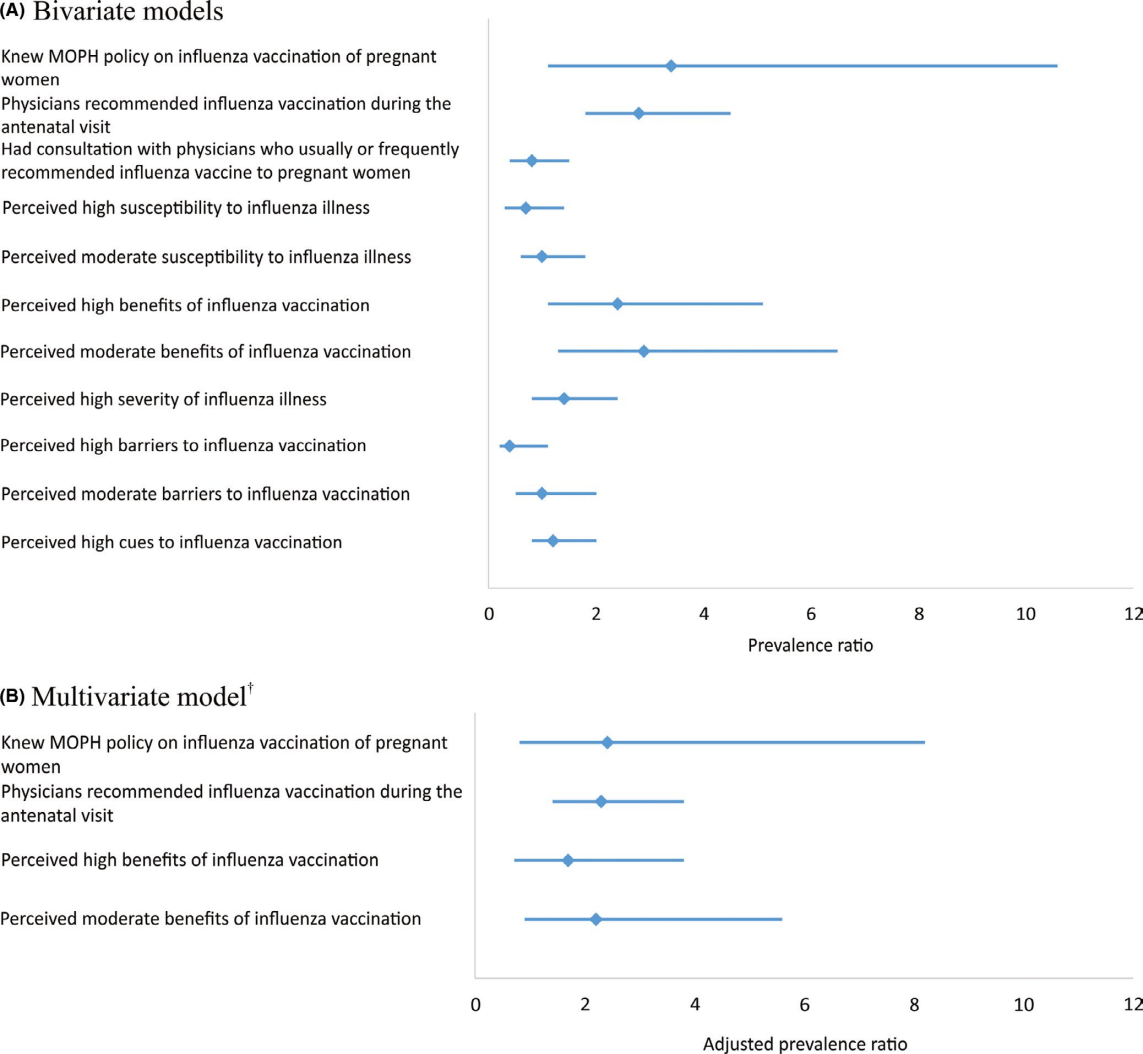
Factors associated with receipt of influenza vaccination by pregnant women. Diamond markers are point estimates, and lines are 95% confidence interval. ^†^Controlling for the pregnant women's knowledge of the recommendation for influenza vaccination in pregnancy and beliefs about the benefits of influenza vaccination

In univariate analysis, the factors significantly associated with influenza vaccination within 30 days of the ANC visit included knowledge of the MOPH policy on influenza vaccination by the pregnant woman (prevalence ratio [PR] 3.4, 95% CI 1.1‐10.6), physician recommendation (PR 2.8; 95% CI 1.8‐4.5), and high (PR 2.4; 95% CI 1.1‐5.1) and moderate (PR 2.9; 95% CI 1.3‐6.5) perceptions of the benefits of influenza vaccination (Figure 2). In the multivariable analysis, the interaction term between perceptions of the benefits of influenza vaccination and physician recommendation was not significant and removed. The final adjusted model identified physician recommendation as the only significant association with vaccination (PR 2.3; 95% CI 1.4‐3.8).

## 4 CONCLUSIONS

Although the efforts within Rajavithi Hospital to increase influenza vaccine uptake apparently increased the proportion of pregnant women vaccinated, the vaccination rate among participating pregnant women was low. In multivariable analyzes, physician recommendation was the most important factor associated with influenza vaccine uptake among Thai pregnant women and the women's perceptions about vaccination had no important association with being vaccinated. All physicians who stated they were frequent recommenders were those who agreed with the MOPH policy on influenza vaccination during pregnancy.

Taking into account women's characteristics alone, we found that knowledge of the MOPH policy on influenza vaccination of pregnant women and recommendation from the physicians were strongly associated with influenza vaccine uptake among pregnant women. Unlike findings of a systematic review of studies conducted mostly in the United States and Canada,^38^ we did not find age, employment status, household income, or education level to be important determinants for influenza vaccination. Previous studies also reported race/ethnicity, smoking status, and number of the ANC visits to be associated with influenza vaccine uptake. We, however, did not examine these factors because: All of the participating women were Asian; smoking during pregnancy was not prevalent in Thailand; and the ANC attendance among pregnant women was relatively common as it was provided free of charge as part of the country's standard of care.

Our multivariable analysis taking into account both providers’ and women's characteristics revealed that pregnant women who received a recommendation from their attending physicians were more than twice as likely to receive the vaccine. This finding is consistent with other studies that demonstrated providers’ recommendation to be the most striking influencer with as much as 100‐fold increase in the likelihood of vaccination in pregnant women they cared for.^46^, ^47^, ^48^, ^49^, ^50^, ^51^ We also found that physician self‐report of how frequently they recommended influenza vaccination to pregnant women in the past was not predictive of their recommendation during the visit as reported by the women. There was also no association between self‐vaccination and recommendation for pregnant women to vaccine.

In Thailand, pregnant women are offered a few vaccines during pregnancy (diphtheria toxoid, tetanus toxoid, and influenza vaccine) as well as series of health promotion education. This might affect the women's ability to accurately recall which vaccines are discussed and prescribed which in turn impacts their responses to this specific question. Prior to vaccination, we found that unvaccinated women who later get the vaccine tended to have lower perceived susceptibility compared with those never got unvaccinated in the concurrent year. In other words, a higher proportion of unvaccinated women believed that they were at risk of influenza infection or exposure compared with those who then unvaccinated, but later got the vaccine, which seems illogical. This might be explained that when translated into Thai language, the “influenza illness” and “common cold” were near homophones, possibly leading to confusion among unvaccinated women who tended to be less knowledgeable about influenza.

In a recent study conducted in Thailand, Praphasiri et al examined the association of characteristics of obstetricians and gynecologists and their reported practice in recommending influenza vaccine to pregnant women using a mail survey method.^52^ They reported that physicians were more likely to routinely recommend influenza vaccine to pregnant women when they were aware of the MOPH policy of influenza vaccination in pregnancy. Consistently, all of our frequent recommenders stated they agreed with the MOPH policy. Findings from Praphasiri et al and ours suggest that awareness of, and agreement with, the MOPH policy may play an important role in increasing influenza vaccine uptake in pregnancy. Our findings also indicated that the academic society for which physicians relied on for information could provide an appropriate venue to raise awareness. Topics to be emphasized include vaccine safety in both pregnant women and fetuses and adverse effects from the influenza vaccine among pregnant women. Additionally, provision of easy access to vaccination services to pregnant women and inclusion of topic related to influenza vaccination into the health promotion sessions to reduce the time physicians need to discuss this topic may be helpful.

This study has a few strengths. First, it used a well‐established theoretical framework to describe the associations between the components of the HBM and influenza vaccination.^38^, ^40^, ^41^ Second, the study was conducted in an upper middle‐income country, as opposed to other studies conducted in industrialized countries, and used a distinctive design that matched participating women with their providers. We were able to link pregnant women with their physicians and report associations linking vaccine uptake and physicians’ recommendation. Third, our participating women were recruited using a systematic sampling approach, making the process less prone to selection bias. Fourth, we used the ACASI method to reduce social desirability bias as participants may over‐report what they believe to be “good.”

This study also has some limitations. First, this study was conducted in one hospital which might affect the result generalization to larger population. The study hospital, however, was among those that have the highest rate of deliveries in Thailand. Second, health beliefs and knowledge of influenza among the women were collected after physician consultation and may have been affected by the visit. Similarly, the health beliefs and knowledge of influenza among physicians may have been affected by the nationwide and hospital campaigns to increase influenza vaccine uptake. Third, this study was conducted at one point in time and participants’ vaccination status determined during a relatively short follow‐up time, it will likely not reflect the trend of information pertinent to the disease and influenza vaccine that is changed with time as more promotional materials and media communications emerge and more doses of vaccine become available. Lastly, the study only accounted for influenza vaccination within 30 days following the ANC visits as we anticipated most women would have been vaccinated by then and decision to get vaccinated after this time interval may be influenced by other factors.

In Thailand, influenza vaccination among pregnant women is among the highest public health intervention priorities. Nine years after its introduction, in 2018 the vaccine has become easier to obtain since the government has launched a year‐round vaccination campaign targeting pregnant women. Our findings demonstrate that methods to improve vaccination coverage among pregnant women include receipt of a physician recommendation for and offer of influenza vaccination and awareness of healthcare personnel about the MOPH policy toward influenza vaccination in pregnancy.
