# Supplementary material for: Predictors for influenza vaccination among Thai pregnant woman: The role of physicians in increasing vaccine uptake
Source: Influenza Other Respir Viruses. 2019 Aug 16;13(6):582–92. doi: 10.1111/irv.12674 (PMC6800306; doi:10.1111/irv.12674)
Supplement: Supplementary file 1 [file IRV-13-582-s001.docx]

**Supplementary table 1: Questions asked to assess knowledge of pregnant women and physicians**

| **Question** | **Answer choices** |
| --- | --- |
| 1. What are the signs and symptoms of influenza? Please select all that you know. | Fever, Runny nose, Coughing, Sore throat, Muscle and joint pain, Difficulty in breathing, Diarrhea and vomiting, I don’t know |
| 1. People can catch influenza from someone with influenza who coughs or sneezes near them. | Yes, I agree, No, I don’t agree, I don’t know |
| 1. People can catch influenza from someone with influenza who coughs or sneezes near them. | Yes, I agree, No, I don’t agree, I don’t know |
| 1. People can catch influenza by touching their mouth or nose after they touch something that has influenza virus on it. | Yes, I agree, No, I don’t agree, I don’t know |
| 1. People can catch influenza from eating food that is improperly handled and cooked. | Yes, I agree, No, I don’t agree, I don’t know |
| 1. Influenza can be prevented through vaccination. | Yes, I agree, No, I don’t agree, I don’t know |
| 1. The chance of getting mildly sick with influenza is the same for everyone. | True, False, I don’t know |
| 1. The chance of getting severely sick with influenza is the same for everyone. | True, False, I don’t know |
| 1. There are medicines to treat influenza. | True, False, I don’t know |
| 1. People can get well from influenza either with or without treatment. | True, False, I don’t know |
| 1. People can die from influenza. | True, False, I don’t know |

**Supplementary table 2: Questions to assess attitude and belief of pregnant women**^†^

| **Question** | **Health Belief Model Construct** | **Level** | **Unvaccinated women (n=293) N (%)** | **Vaccinated pregnant women (n=42) N (%)** | ***P* value** |
| --- | --- | --- | --- | --- | --- |
| 1. I’m unlikely to get influenza during pregnancy. | Susceptibility | High (Disagree) | 69 (24) | 9 (21) | 0.23 |
|  |  | Moderate (Neither agree or disagree) | 123 (42) | 13 (31) |  |
|  |  | Low (Agree) | 101 (34) | 20 (48) |  |
| 2. Someone in my family or group of friends is likely to get sick with influenza this year.^‡^ | Susceptibility | High (Agree) | 54 (18) | 8 (19) | 0.70 |
|  |  | Moderate (Neither agree or disagree) | 131 (45) | 16 (38) |  |
|  |  | Low (Disagree) | 108 (37) | 18 (43) |  |
| 3. The influenza vaccine can lower pregnant women’s risk of getting influenza.^‡^ | Benefit | High (Agree) | 216 (74)^*^ | 37 (88)^*^ | 0.12 |
|  |  | Moderate (Neither agree or disagree) | 74 (25)^*^ | 5 (12)^*^ |  |
|  |  | Low (Disagree) | 3 (1)^*^ | 0 (0)^*^ |  |
| 4. If I get influenza vaccination during pregnancy, the vaccine is likely to prevent my baby from getting influenza when he/she is born.^‡^ | Benefit | High (Agree) | 181 (62)^*^ | 32 (76)^*^ | 0.12 |
|  |  | Moderate (Neither agree or disagree) | 99 (34)^*^ | 10 (24)^*^ |  |
|  |  | Low (Disagree) | 13 (4)^*^ | 0 (0)^*^ |  |
| 5. The influenza vaccine can prevent pregnant women from getting sick with influenza.^‡^ | Benefit | High (Agree) | 220 (75)^*^ | 35 (83)^*^ | 0.42 |
|  |  | Moderate (Neither agree or disagree) | 68 (23)^*^ | 7 (17)^*^ |  |
|  |  | Low (Disagree) | 5 (2)^*^ | 0 (0)^*^ |  |
| 6. If I get influenza during pregnancy, it may be harmful to my baby.^‡^ | Severity | High (Agree) | 225 (77) | 33 (78) | 0.29 |
|  |  | Moderate (Neither agree or disagree) | 54 (18) | 5 (12) |  |
|  |  | Low (Disagree) | 14 (5) | 4 (10) |  |
| 7. If I get influenza during pregnancy, it is likely to be more severe or the chance of having a complication may be higher than other people.^‡^ | Severity | High (Agree) | 223 (76) | 33 (79) | 0.05 |
|  |  | Moderate (Neither agree or disagree) | 57 (19) | 4 (9) |  |
|  |  | Low (Disagree) | 13 (4) | 5 (12) |  |
| 8. My doctor’s recommendation is an important consideration in my decision to get influenza vaccine.^‡^ | Cue to action | High (Agree) | 221 (75)^*^ | 37 (88)^*^ | 0.17 |
|  |  | Moderate (Neither agree or disagree) | 66 (23)^*^ | 5 (12)^*^ |  |
|  |  | Low (Disagree) | 6 (2)^*^ | 0 (0)^*^ |  |
| 9. My family’s recommendation is an important consideration in my decision to get influenza vaccine.^‡^ | Cue to action | High (Agree) | 159 (54)^*^ | 25 (59)^*^ | 0.81 |
|  |  | Moderate (Neither agree or disagree) | 118 (40)^*^ | 15 (36)^*^ |  |
|  |  | Low (Disagree) | 16 (6)^*^ | 2 (5)^*^ |  |
| 10. I had an allergic reaction to a vaccination in the past (could be any type of vaccine).^‡^ | Barrier | High (Agree) | 60 (21) | 8 (19) | 0.90 |
|  |  | Moderate (Neither agree or disagree) | 115 (39) | 18 (43) |  |
|  |  | Low (Disagree) | 118 (40) | 16 (38) |  |
| 11. The influenza vaccine is safe for me during pregnancy. | Barrier | High (Disagree) | 17 (6) | 0 (0)^*^ | 0.03 |
|  |  | Moderate (Neither agree or disagree) | 112 (38) | 10 (24)^*^ |  |
|  |  | Low (Agree) | 164 (56) | 32 (76)^*^ |  |
| 12. The influenza vaccine is safe for my baby during pregnancy. | Barrier | High (Disagree) | 12 (4) | 1 (2)^*^ | 0.09 |
|  |  | Moderate (Neither agree or disagree) | 109 (37) | 9 (21)^*^ |  |
|  |  | Low (Agree) | 172 (59) | 32 (76)^*^ |  |
| 13. I’m willing to receive an influenza vaccination during pregnancy even if I have to spend some extra time or do extra step during the hospital visit. | Barrier | High (Disagree) | 18 (6) | 1 (2)^*^ | 0.01 |
|  |  | Moderate (Neither agree or disagree) | 117 (40) | 8 (19)^*^ |  |
|  |  | Low (Agree) | 158 (54) | 33 (79)^*^ |  |

^†^3-point scale: Do you agree or disagree with each of the following statement? (1) agree, (2) neither agree nor disagree, and (3) disagree

^‡^Item was reverse coded so that higher values reflect higher levels of the Health Belief Model construct.

^*^*P* value<0.01 for linear trend within each element.

**Supplementary table 3: Questions to create Health Belief Model-like constructs for physicians**^†^

| **Question** | **Health Belief Model Construct** | **Level** | **Frequent recommenders**^‡^  **(n=13) N (%)** | **Non-frequent recommenders**^‡^  **(n=26) N (%)** | ***P* value** |
| --- | --- | --- | --- | --- | --- |
| 1. My pregnant patient is at risk of getting influenza during her pregnancy.^§^ | Susceptibility | High (Agree) | 11 (85)^*^ | 23 (88)^*^ | 1.00 |
|  |  | Moderate (Neither agree or disagree) | 2 (15)^*^ | 3 (12)^*^ |  |
|  |  | Low (Disagree) | 0 (0)^*^ | 0 (0)^*^ |  |
| 2. The influenza vaccine can prevent pregnant women from getting sick with influenza.^§^ | Benefit | High (Agree) | 11 (85)^*^ | 22 (85)^*^ | 1.00 |
|  |  | Moderate (Neither agree or disagree) | 2 (15)^*^ | 4 (15)^*^ |  |
|  |  | Low (Disagree) | 0 (0)^*^ | 0 (0)^*^ |  |
| 3. If a pregnant woman gets influenza vaccination during her pregnancy, the vaccination will provide protection against influenza to her newborn while he or she is less than six months of age.^§^ | Benefit | High (Agree) | 13 (100)^*^ | 21 (81)^*^ | 0.38 |
|  |  | Moderate (Neither agree or disagree) | 0 (0)^*^ | 4 (15)^*^ |  |
|  |  | Low (Disagree) | 0 (0)^*^ | 1 (4)^*^ |  |
| 4. Pregnant women have an increased risk of getting severely ill from influenza compared to other women.^§^ | Severity | High (Agree) | 12 (92) | 19 (73)^*^ | 0.48 |
|  |  | Moderate (Neither agree or disagree) | 0 (0) | 4 (15)^*^ |  |
|  |  | Low (Disagree) | 1 (8) | 3 (12)^*^ |  |
| 5. The Ministry of Public Health’s recommendations related to influenza vaccination are an important consideration in my decision about whether to recommend influenza vaccine to my pregnant patient.^§^ | Cue to action | High (Agree) | 12 (92)^*^ | 22 (85)^*^ | 0.65 |
|  |  | Moderate (Neither agree or disagree) | 1 (8)^*^ | 4 (15)^*^ |  |
|  |  | Low (Disagree) | 0 (0)^*^ | 0 (0)^*^ |  |
| 6. My hospital’s or clinic’s policy on influenza vaccination is an important consideration in my decision about whether to recommend influenza vaccine to my pregnant patient | Cue to action | High (Disagree) | 0 (0)^*^ | 1 (4)^*^ | 1.00 |
|  |  | Moderate (Neither agree or disagree) | 1 (8)^*^ | 3 (11)^*^ |  |
|  |  | Low (Agree) | 12 (92)^*^ | 22 (85)^*^ |  |
| 7. The Royal Thai College of the Obstetricians and Gynaecologists’ recommendation on influenza vaccination is an important consideration in my decision about whether to recommend influenza vaccine to my pregnant patient.^§^ | Cue to action | High (Agree) | 13 (100)^*^ | 22 (85)^*^ | 0.28 |
|  |  | Moderate (Neither agree or disagree) | 0 (0)^*^ | 4 (15)^*^ |  |
|  |  | Low (Disagree) | 0 (0)^*^ | 0 (0)^*^ |  |
| 8. The influenza vaccine is safe for pregnant women during their pregnancies. | Barrier | High (Disagree) | 0 (0) | 0 (0) | 0.08 |
|  |  | Moderate (Neither agree or disagree) | 0 (0)^*^ | 6(23)^*^ |  |
|  |  | Low (Agree) | 13 (100)^*^ | 20 (77)^*^ |  |
| 9. Influenza vaccination of pregnant women is safe for the developing fetus. | Barrier | High (Disagree) | 0 (0)^*^ | 0 (0)^*^ | 0.08 |
|  |  | Moderate (Neither agree or disagree) | 0 (0)^*^ | 6 (23)^*^ |  |
|  |  | Low (Agree) | 13 (100)^*^ | 20 (77)^*^ |  |
| 10. Pregnant women may have adverse effects from the influenza vaccine.^§^ | Barrier | High (Agree) | 7 (54)^*^ | 17 (65)^*^ | 0.76 |
|  |  | Moderate (Neither agree or disagree) | 5 (38) | 7 (27)^*^ |  |
|  |  | Low (Disagree) | 1 (8) | 2 (8)^*^ |  |
| 11. If my pregnant patient had to spend some extra time or do an extra step during her hospital visit to get the influenza vaccine, I would not recommend that my patient get the influenza vaccination.^§^ | Barrier | High (Agree) | 0 (0)^*^ | 3 (12)^*^ | 0.55 |
|  |  | Moderate (Neither agree or disagree) | 2 (15)^*^ | 6 (23)^*^ |  |
|  |  | Low (Disagree) | 11 (85)^*^ | 17 (65)^*^ |  |
| 12. The amount of time I have for a consultation with my pregnant patient is an important consideration when deciding whether to recommend influenza vaccine to my pregnant patient.^§^ | Barrier | High (Agree) | 4 (31) | 9 (35) | 0.61 |
|  |  | Moderate (Neither agree or disagree) | 4 (31) | 4 (15) |  |
|  |  | Low (Disagree) | 5 (38) | 13 (50) |  |

^†^5-point scale: Do you agree or disagree with each of the following statement? (1) strongly agree, (2) agree (3) neither agree nor disagree (4) disagree and (5) strongly disagree. Scale was reduced by assigning 1 point to either “strongly agree” or “agree,” two points to “neither agree nor disagree,” and three points to “disagree” or “strongly disagree”.

^‡^frequent recommenders were those reported recommending influenza vaccination to ≥50% of eligible pregnant women; non-frequent recommenders were those reported recommending influenza vaccination to <50% of eligible pregnant women.

^§^Item was reverse coded so that higher values reflect higher levels of the Health Belief Model construct.

^*^*P* value<0.01 for linear trend within each element.

**Supplementary table 4. Health Belief Model constructs divided into two (low, high) or three categories (low, moderate, high).**^†^

|  | Score |
| --- | --- |
| Susceptibility to influenza  High  Moderate  Low | 5 and 6 points  4 points  0 to 3 points |
| Benefits of influenza vaccination  High  Moderate  Low | 9 points  8 points  0 to 7 points |
| Severity of influenza illness  High  Low | 6 points  0 to 5 points |
| Barriers to influenza vaccination  High  Moderate  Low | 4 to 5 points  6 to 7 points  0 to 5 points |
| Cues to action to influenza vaccination  High  Low | 6 points  0 to 5 points |

^†^Scores from individual questions within the same construct were summed and used as the score for that construct.

**Supplementary table 5. Characteristics and Health Belief Model constructs of pregnant women**

| **Variables** | **All pregnant women (n=610)**  **N (%)** | **Unvaccinated women matched to a physician  (n=335)**  **N (%)** | ***P* value** |
| --- | --- | --- | --- |
| Age, median (IQR) | 27 (23-33) | 27 (23-33) | 0.42 |
| Trimester  Second  Third | 266 (44)  344 (56) | 172 (51)  163 (49) | 0.02 |
| Pre-existing condition | 21 (3) | 17 (5) | 0.12 |
| Employed outside home | 432 (71) | 232 (69) | 0.35 |
| Household income ≥30,000 Baht/month (876 US dollars)^†^ | 208 (34) | 110 (33) | 0.76 |
| Completed secondary school or higher | 428 (70) | 244 (73) | 0.52 |
| High level knowledge of influenza^‡^ | 206 (34) | 108 (32) | 0.53 |
| Knew of MOPH policy on influenza vaccination of pregnant women^§^ | 515 (84) | 279 (83) | 0.69 |
| Saw promotional material at or before enrollment visit | 521 (85) | 281 (84) | 0.68 |
| Vaccinated against influenza prior to enrollment | 89 (15) | NA |  |
| Physician recommended influenza vaccine^‡^ | 210/521 (40) | 140 (42) | 0.56 |
| Health Belief Model constructs  Susceptibility to influenza  High  Moderate  Low  Benefits of influenza vaccination  High  Moderate  Low  Severity of influenza illness  High  Low  Barriers to influenza vaccination  High  Moderate  Low  Cues to action to influenza vaccination  High  Low | 136 (22)  225 (37)  249 (41)  343 (56)  122 (20)  145 (24)  435 (71)  175 (29)  126 (21)  359 (59)  125 (20)  329 (54)  281 (46) | 68 (20)  131 (39)  136 (41)  183 (55)  63 (19)  89 (27)  231 (69)  104 (31)  81 (24)  190 (57)  64 (19)  170 (51)  165 (49) | 0.71  0.63  0.45  0.45  0.35 |

NA, not applicable; MOPH, Ministry of Public Health

^†^Among those who answered this question.

^‡^Scored at least 9 correct answers out of 11 questions asked on symptoms, modes of transmission, and treatment of influenza, benefits of vaccination, and high-risk groups recommended for influenza vaccination.

^§^Among those not already vaccinated. Sample sizes are indicated (n/N).

**Supplementary table 6. Characteristics and Health Belief Model constructs of antenatal care clinic physicians**^†^

| **Variables** | **All physicians**  **(n=60)  N (%)** | **Physicians matched to a pregnant woman (n=39)**  **N (%)** | ***P* value** |
| --- | --- | --- | --- |
| Age, median (IQR) | 30 (28-33) | 31 (28-35) | 0.38 |
| Years of practice, median (IQR) | 5 (4-8) | 5 (4-10) | 0.44 |
| Female | 50 (83) | 31 (79) | 0.62 |
| High level of knowledge of influenza^‡^ | 41 (68) | 23 (59) | 0.36 |
| Agreed with MOPH policy on influenza vaccination of pregnant women | 53 (88) | 34 (87) | 0.88 |
| Reported frequency of recommendation of influenza vaccine to pregnant women  Frequently, usually, or always  Occasionally, sometimes  Never or rarely | 17 (28)  27 (45)  16 (27) | 13 (33)  20 (51)  6 (15) | 0.42 |
| Health Belief Model constructs regarding perceptions of pregnant women  Susceptibility to influenza  High  Moderate  Low  Benefits of influenza vaccination  High  Moderate  Low  Severity of influenza illness  High  Moderate  Low  Barriers to vaccination  High  Moderate  Low  Cues to action to recommend influenza vaccination  High  Moderate  Low | 54 (90)  0 (0)  6 (10)  42 (70)  0 (0)  18 (30)  47 (78)  0 (0)  13 (22)    23 (38)  11 (18)  26 (43)  52 (87)  0 (0)  8 (13) | 34 (87)  0 (0)  5 (13)  26 (67)  0 (0)  13 (33)  31 (79)  0 (0)  8 (21)  16 (41)  7 (18)  16 (41)  32 (82)  0 (0)  7 (18) | 0.66  0.73  0.89  0.96  0.53 |

IQR, interquartile range; MOPH, Ministry of Public Health

^†^Percentages are proportions of non-missing data.

^‡^Scored at least 9 correct answers out of 11 questions asked on symptoms, modes of transmission, and treatment of influenza, benefits of vaccination, and high-risk groups recommended for influenza vaccination.
